# Bioadhesive polymer/lipid hybrid nanoparticles as oral delivery system of raloxifene with enhancive intestinal retention and bioavailability

**DOI:** 10.1080/10717544.2021.1872742

**Published:** 2021-01-27

**Authors:** Xinhui Du, Na Gao, Xiaoyong Song

**Affiliations:** a Out-patient Department, Huaihe Hospital Affiliated to Henan University, Kaifeng, China; b Department of Pharmaceutics, People's Hospital of Jinming District, Kaifeng, China; c School of Pharmacy, Henan University, Kaifeng, China

**Keywords:** Raloxifene, hybrid nanoparticles, bioadhesion, bioavailability, intestinal retention

## Abstract

Raloxifene (RLX) is a second-generation selective estrogen receptor modulator used to treat osteoporosis in postmenopausal women. RLX fails to be developed into injectable dosage forms due to poor solubility. Although oral formulations are clinically available, the lower bioavailability (<2%) embarrasses the pharmaceutists. This work reported a bioadhesive nanosystem intended for oral delivery of RLX to enhance its oral bioavailability and address the formulation challenge. The bioadhesive nanosystem refers to polymer–lipid hybrid nanoparticles made up of Carbopol 940, glyceryl distearate, and TGPS. RLX was solidly encapsulated into bioadhesive nanoparticles (*b*NPs) through a nanoprecipitation technique along with synchronous desalting of RLX·HCl. The resultant RLX-loaded *b*NPs (RLX-*b*NPs) were characterized by particle size, *ζ* potential, morphology, and entrapment efficiency. The *in vitro* release and *in vivo* oral bioavailability of RLX-*b*NPs in rats were comparatively investigated with RLX-loaded common lipid nanoparticles (RLX-*c*NPs). The preferred formulation possesses a particle size of 150 nm around with a polydispersity index (PDI) of 0.282. RLX-*b*NPs exhibited slower drug release than RLX-*c*NPs owing to the presence of an adhesive layer. After oral administration, RLX-*b*NPs resulted in significant enhancement in the bioavailability of RLX, up to 556.9% relative to RLX suspensions, while it was merely 244.7% for RLX-*c*NPs. Cellular testing and *ex vivo* transport imaging demonstrated that *b*NPs were endowed with excellent intestinal epithelial affinity and absorbability. Our study affords an alternative option for designing a suitable oral delivery system specific to amphiphobic drugs like RLX·HCl.

## 1. Introduction

Raloxifene (RLX) is a type of drug called selective estrogen receptor modulator (SERM) used to treat osteoporosis in postmenopausal women and estrogen-dependent breast cancer (Ko & Jordan, 2011). The oral bioavailability of RLX reported to be less than 2% due to first-pass effect and poor solubility (Hochner-Celnikier, 1999). The chemical form of RLX available on the market is its hydrochloride (RLX·HCl). Despite being processed into hydrochloride, its solubility is still unmet, not only poorly soluble in water but also in oil (i.e. amphiphobic), resulting in a great challenge in formulation. To overcome the undesirable physiochemical properties, a variety of formulation strategies have been explored for optimizing the oral delivery of RLX·HCl, including lipid-based nanoparticles (Ravi et al., 2014; Izgelov et al., 2018; Soni et al., 2020), mesoporous carbon nanospheres (Ye et al., 2016), microemulsions (Shah et al., 2018), inclusion complexes-based nanoparticles (Wang & Li, 2018), and nanomicelles (Varshosaz et al., 2019). To varying degrees, these systems improved the oral bioavailability of RLX·HCl. However, previous studies paid less attention to the issues of loading stability and intestinal transport time. The hydrophilicity of hydrochloride makes RLX·HCl easily precipitate from nanoparticles. In addition, common nanocarriers are rarely featured with a longish intestinal retention. Therefore, it is highly encouraged to develop a novel carrier system to ulteriorly improve the oral delivery of RLX.

Polymer–lipid hybrid nanoparticles made up of a functional polymer and a biocompatible lipid are emerging as nanodrug carriers that integrate the beneficial features of both polymeric and lipidic nanocarriers (Rao & Prestidge, 2016; Maghrebi et al., 2019). Different from single polymeric nanoparticles (Jahangir et al., 2018), the lipid component facilitates stable encapsulation for drug, while the polymer provides functionalities for nanoparticles, such as enhancive stability and accessorial bioadhesion. Plenty of studies have demonstrated the superiority of polymer–lipid hybrid systems to single-component counterparts in formulation stability, drug loading, release regulation, cellular uptake, and intestinal absorption (Varthya et al., 2016; Hallan et al., 2017; Dalmoro et al., 2018; Gou et al., 2018; Ren et al., 2018; Ana et al., 2019; Yin et al., 2019). In terms of oral polymer–lipid hybrid nanoparticles, the introduction of a polymer with low digestibility or degradability reinforces the gastrointestinal stability of nanocarriers (Yin et al., 2016; Joyce et al., 2017), which is favorable for preventing drug precipitation from carriers upon digestion. As known, the absorption amount of a drug is proportional to the transport time of its preparations in the gastrointestinal tract. At this point, bioadhesive polymers have considerable advantage over nonadhesive ones in potentiating the performance of oral nanoparticles. On the one hand, the bioadhesive polymer intensifies the physiological stability of lipid-containing nanoparticles; on the other hand, it renders the carrier a bioadhesive trait. These two factors jointly prolong the gastrointestinal transport time and drug absorption duration. A bioadhesive polymer–lipid hybrid system should be more promising in ameliorating the oral bioavailability of RLX.

In this study, a bioadhesive polymer–lipid hybrid system based on carbomer, glyceryl distearate, and TGPS was developed for oral delivery of RLX. RLX-loaded bioadhesive polymer–lipid hybrid nanoparticles (RLX-*b*NPs) were prepared through a nanoprecipitation technique upon synchronous desalting of RLX·HCl. We characterized RLX-*b*NPs with particle size, *ζ* potential, morphology, and entrapment efficiency. The *in vitro* drug release and *in vivo* bioavailability were investigated followed by cellular and bioadhesive evaluations.

## 2. Materials and methods

### 2.1. Materials

Raloxifene hydrochloride (RLX·HCl), d-α-tocopheryl polyethylene glycol 1000 succinate (TPGS) and 3,3′-dioctadecyloxacarbocyanine perchlorate (DiO) were purchased from Aladdin Bio-Chem Technology Co., Ltd. (Shanghai, China). Carbomer (Carbopol 940) was provided by Nanjing Well Pharmaceutical Co., Ltd. (Nanjing, China). Glyceryl distearate (Precirol ATO 5) was kindly gifted by Gattefosse (Saint-Priest Cedex, France). All other chemicals were of analytical grade and used as received.

### 2.2. Preparation of RLX-*b*NPs

RLX-*b*NPs were prepared through a nanoprecipitation technique with a minor modification (Du et al., 2019; Li et al., 2019). First, RLX·HCl was dissolved in 75% ethanol solution containing an appropriate amount of NaOH. The alkalic solution resulted in desalting of RLX·HCl and generated hydrophobic RLX. Then, Precirol ATO 5, Carbopol 940, and TGPS were introduced into the solution followed by heating at 55 °C. After all ingredients have been dissolved, the solution was slowly injected into water under agitation (1000 rpm) for self-assembly. Upon diffusion of ethanol into water, RLX precipitated with other components and formed RLX-*b*NPs due to solubility plummet. After that, the residual solvent was removed by evaporation under a reduced pressure. The factors influencing the formulation properties (particle size and EE) were investigated by altering one variable and fixing others, including the ratio of RLX to excipients and the ratio of organic to aqueous phase. In addition, RLX-loaded common lipid nanoparticles (RLX-*c*NPs), as a reference formulation, were prepared following the same procedure using Precirol ATO 5 alone.

### 2.3. Characterization of nanoparticles

The particle size and *ζ* potential of RLX-*b*NPs and RLX-*c*NPs were measured using a laser particle analyzer (Zetasizer Nano ZS, Malvern, Worcestershire, UK) at 25 °C. The samples were diluted 50 times around with deionized water and then proceeded to laser diffraction or electrophoretic mobility. The particle size and *ζ* potential were output from the build-in software based on the principles of dynamic light scattering and Doppler velocimetry. The morphology of RLX-*b*NPs was observed by a JEM-1200EX transmission electron microscope (TEM) (JEOL, Tokyo, Japan). The samples were properly diluted and directly inspected under TEM after fixation. TEM micrographs were taken with CCD camera at the acceleration voltage of 100 kV.

### 2.4. Determination of EE

The determination of EE referred to the reported procedure (Li et al., 2019). RLX-*b*NPs were first centrifuged at 5000 rpm for 5 min to remove the possibly unloaded RLX. Afterward, the upper clear nanosuspensions were withdrawn and placed in a centrifugal filter device (Amicon^®^ Ultra-0.5, MWCO 50 kDa, Merck, Darmstadt, Germany). The samples were then subjected to centrifugation at 10,000 rpm for 10 min to collect the filtrate. RLX concentration in the filtrate was analyzed by HPLC established below. EE was calculated using the equation: EE (%)=(1 – *M*_fre_/*M*_tot_)×100%, where *M*_fre_ and *M*_tot_ denote the amount of free RLX in RLX-*b*NPs and total RLX in the system, respectively.

Chromatographic assay on RLX was performed by an Agilent 1200 Series HPLC system (Agilent, Santa Clara, CA) equipped with an UV detector. The samples were eluted against a Thermo Syncronis C_18_ column (5 μm, 4.6 mm × 250 mm) at 40 °C with an injection volume of 20 μL. The mobile phase consisted of 35% acetonitrile and 65% water adjusted to pH 3.0 with formic acid that was pumped at a flow rate of 1.0 mL/min (Ye et al., 2016). The signals of eluents were collected at 288 nm.

### 2.5. *In vitro* release study

The *in vitro* release of RLX-*c*NPs and RLX-*b*NPs were carried out in water, 0.1 M HCl, and pH 6.8 PBS using a reverse dialysis technique (Zhang et al., 2015). To create a sink condition, Tween 80 (1%, g/w) was added into the release medium as a solubilizing agent. Aliquots of samples equal to 25 mg of RLX were added into the dissolution cups loading 250 mL of release medium. Meanwhile, the ready-to-use dialysis tubes (MWCO 100 kDa) loading blank medium were placed in the dissolution cups. Dialysis was performed at 37 °C under stirring of 100 rpm. At 0.25, 0.5, 1, 2, 4, 8, 10, and 12 h, 200 μL of release solution were withdrawn from the tube. The concentration of RLX in the dialysates was determined by HPLC as described above. The accumulative release percentages of RLX from nanoparticles in three different kinds of media were calculated, and the release profiles were plotted accordingly.

### 2.6. Bioavailability study in rats

SD rats (220 ± 20 g) were fasted overnight prior to administration but allowed access to water *ad lib*. The rats were randomly grouped into three groups (*n*= 6), i.e. RLX suspensions (dispersed in 0.5% CMC–Na solution), RLX-*c*NPs, and RLX-*b*NPs. Then, the rats were given with RLX preparations at the dose of 25 mg/kg by gavage. At the predetermined intervals (0.5, 1, 2, 4, 6, 8, 12, 16, and 24 h), approximately 0.25 mL of blood were withdrawn from the caudal vein and transferred into heparinized tubes. The blood samples were immediately centrifuged at 5000 rpm for 5 min to collect the plasma. The protocols for animal experiment were reviewed and approved by the Experimental Animal Ethical Committee of Henan University.

The resulting plasmas were deproteinized with fourfold volume of acetonitrile (Yin et al., 2017c). The plasma samples were mixed with acetonitrile and eddied for 60 s. After vortex, the mixtures were centrifuged at 10,000 rpm for 5 min to collect the supernatants. RLX in the supernatants was quantified by HPLC as described above with oridonin as an internal standard. The pharmacokinetic data were processed using a freely available program, PK*Solver* 2.0.

### 2.7. Cellular uptake and internalization

Caco-2 cells were used to evaluate the cellular uptake of free RLX (solution), RLX-*c*NPs, and RLX-*b*NPs. Caco-2 cells from ATCC were cultured in DMEM supplemented with 10% fetal bovine serum (FBS) and 100 IU/mL of penicillin–streptomycin solution at 37 °C in a humidified atmosphere of 5% CO_2_. For the cellular uptake experiment, the cells were seeded in 12-well plate at a density of 1 × 10^5^ cells/well and cultured for 24 h. Next, the culture medium was discarded and replaced by DMEM-diluted RLX, RLX-*c*NPs, or RLX-*b*NPs (50 μg/mL). The cells were incubated with the medications for 0.5, 1, and 2 h at 37 °C, respectively. After washing twice with pH 7.4 HBSS, the cells were lysed by RIPA lysis buffer. After vortex for 3 min, the lysates were centrifuged at 12,000 rpm for 5 min under 4 °C to collect the supernatant. RLX in the supernatant was then analyzed by HPLC.

Another batch of well-cultured cells was used to observe the cellular internalization of RLX-*c*NPs and RLX-*b*NPs. Caco-2 cells were cultured in a six-well plate for 24 h, in which microslides were placed in advance. Subsequently, the cells were incubated with DiO-labeled RLX-*c*NPs and RLX-*b*NPs at 37 °C for 0.5 h. Afterwards, the cells were washed twice with cold HBSS and fixed with 4% paraformaldehyde for 1 h. The internalization of nanoparticles into Caco-2 cells was observed using a LSM510 CLSM (Zeiss, Oberkochen, Germany) after nucleus staining with Hoechst 33258.

### 2.8. Bioadhesion evaluation

Bioadhesion of RLX-*b*NPs was evaluated by a modified mucin particle method (Takeuchi et al., 2005) and *ex vivo* imaging of intestinal transport (Deng et al., 2019). To prepare the mucin particles, 1 g of mucin powders from the porcine stomach and intestine were added into 100 mL of saline and subjected to stirring for 12 h. The coarse mucin particles were then incubated overnight at 37 °C. The mucin suspensions with a mean particle size of ∼300 nm were prepared by sonication at 300 W for 15 min. After centrifugation at 5000 rpm for 10 min, a supernatant containing homogeneous submicron mucin particles was obtained. Then, an aliquot of RLX-*c*NPs or RLX-*b*NPs (0.5 mL) was added to 1.5 mL of mucin suspensions and continuously incubated for 0.5, 1, and 2 h at 37 °C. Then, the change in particle size and *ζ* potential pertinent to the system were measured.

To investigate the intestinal retention of different nanocarriers, DiO-labeled RLX-*c*NPs and RLX-*b*NPs were freshly prepared. SD rats fasted for two days were orally administrated with DiO-labeled RLX-*c*NPs and RLX-*b*NPs. The rats were sacrificed by cervical dislocation 1 h after administration. Afterwards, the small intestine of rats was taken out, and a length of the duodenum, jejunum, and ileum was cut off, respectively. The intestinal segments were rinsed using cool saline for twice followed by fixation with 4% paraformaldehyde. Then, the intestinal segments were made into paraffin slices and stained with DAPI. The fluorescence staining of the absorptive epithelium by RLX-*c*NPs and RLX-*b*NPs was appreciated using CLSM.

## 3. Results and discussion

### 3.1. Preparation and characterization of RLX-*b*NPs

**Figure 1.**
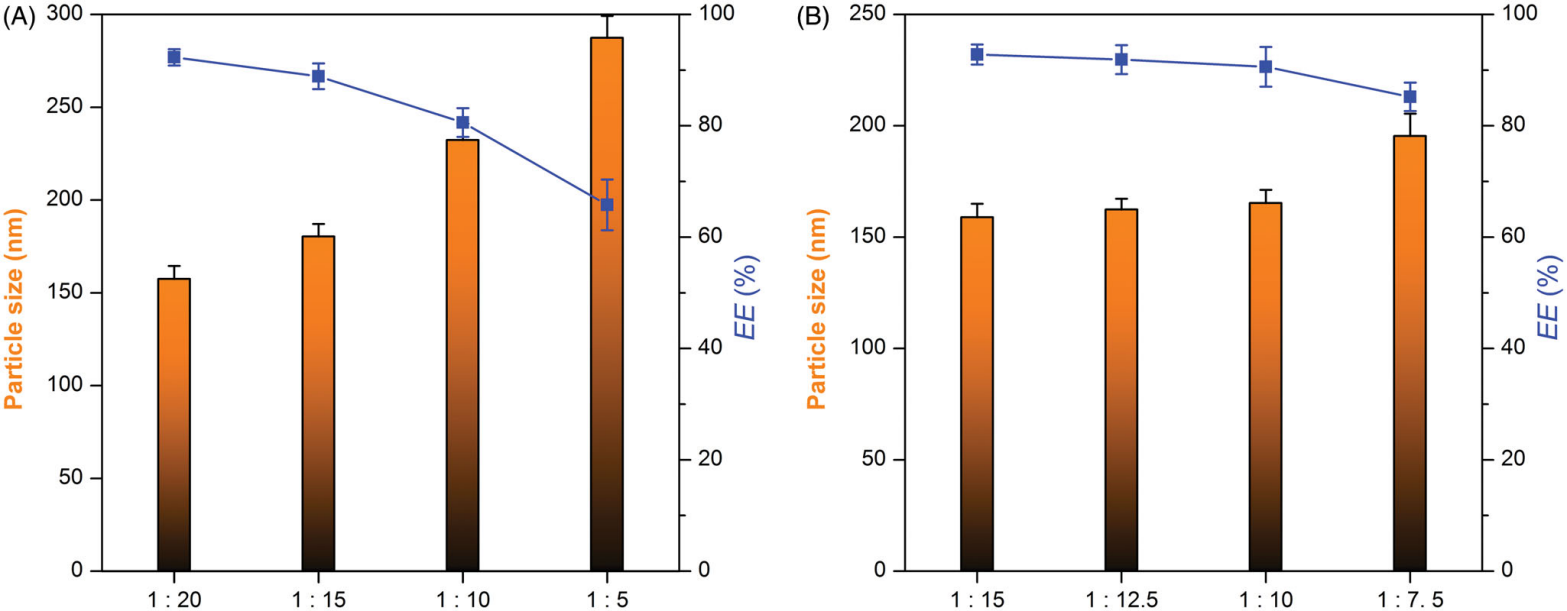
Effects of RLX/excipients ratio (A) and organic/aqueous phase ratio (B) on particle size and EE of nanoparticles. Lipidic components include Precirol ATO 5, Carbopol 940, and TGPS in a fixed ratio of 2:2:1.

The great challenge of formulating RLX·HCl into nanoparticles lies in its amphiphobicity that tends to result in precipitation after encapsulation due to immiscibility with both water and oil or lipid. In this study, we dissolved RLX·HCl in an alkalic cosolvent to desalinate it and then encapsulated free base into nanoparticles by a solvent diffusion technique. The formulation factors influencing the performance of nanoparticles are shown in Figure 1. The ratio of drug to excipients had great effects on the particle size and EE of RLX-*b*NPs. The particle size of nanoparticles increased with increase of the ratio of drug to excipients, while the EE moved toward the opposite direction. A lower ratio of drug to excipients in the formulation would be beneficial for particle size reduction and EE augment. It indicates that more drug molecules affect the self-assembly of polymer and lipid under the action of TPGS due to its high melting point. The organic/water phase ratio upon preparation showed marginal effects on the particle size and EE of nanoparticles below the ratio of 1/7.5. Nevertheless, a lower ratio of organic to water phase was also advantageous to produce smaller RLX-*b*NPs with a high EE. This may be related to the diffusion velocity of ethanol toward water. At a low concentration, carrier materials tend to self-assemble quickly and thus encapsulate the drug immediately. Considering the advantages of small particle size and high EE in oral drug delivery, the formulation was finalized as 10 mg of RLX (base), 60 mg of Precirol ATO 5, 60 mg of Carbopol 940, and 30 mg of TGPS that were formulated into 20 mL of water using 2 mL of 75% ethanol.

**Figure 2.**
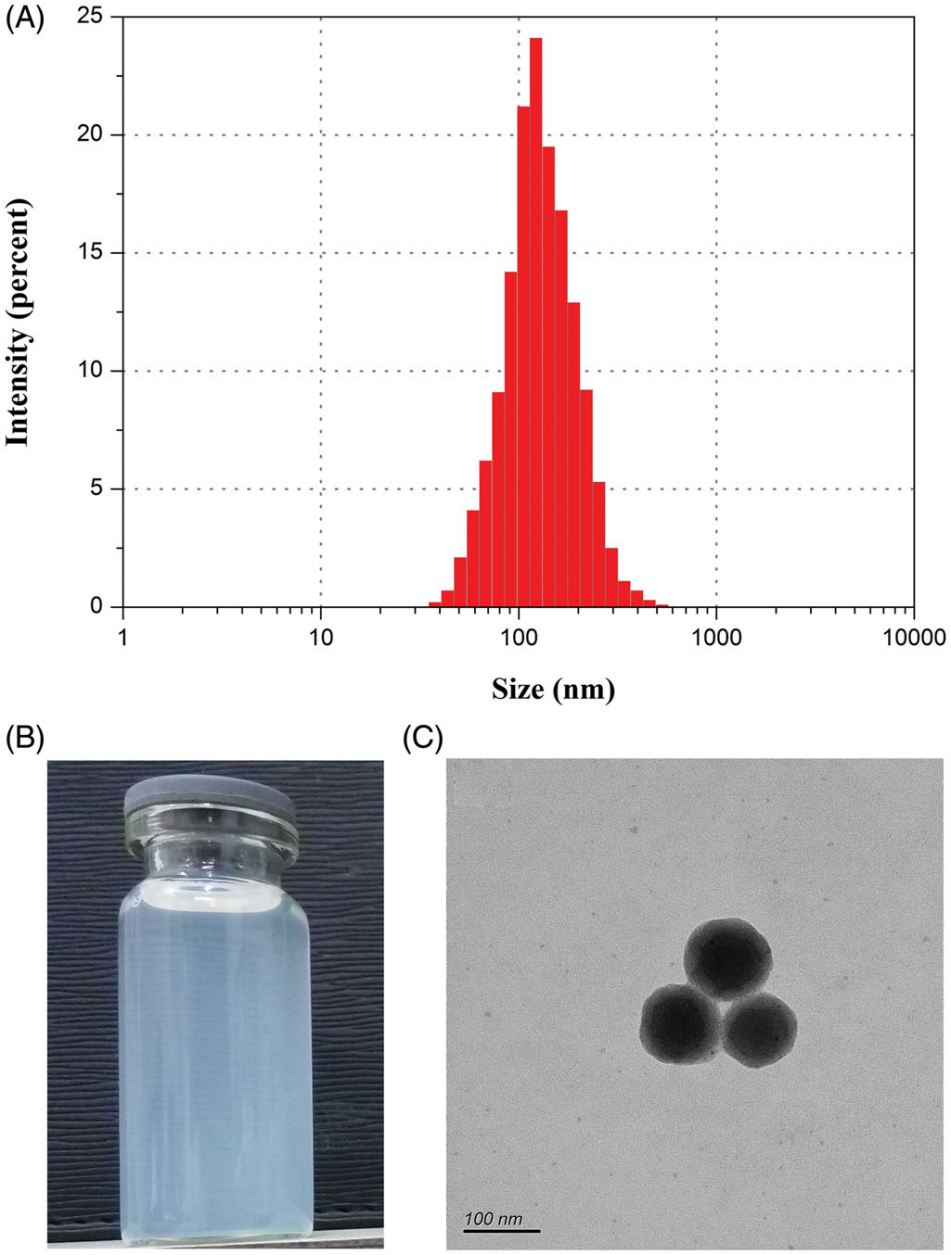
Characterization of RLX-bNPs: particle size distribution (A), appearance (B), and TEM morphology (C).

RLX-*b*NPs prepared from the final formulation were 156 nm in particle size with a polydispersity index (PDI) of 0.282 (Figure 2(A)). The nanosuspension of RLX-*b*NPs showed a transparent blue appearance (Figure 2(B)). RLX-*b*NPs were spherical in morphology as revealed by TEM (Figure 2(C)). The particle size present in TEM was slightly smaller than the hydrodynamic size measured based on the dynamic light scattering. This can be explained by dehydration of nanoparticles, especially when there is a bioadhesive layer around the nanoparticles. The EE of RLX-*b*NPs was measured to be 94.47%, demonstrating a high drug loading. In addition, RLX-*b*NPs were negatively charged with a *ζ* potential of −36.2 mV. The absolute *ζ* potential over 25 mV also suggests that the prepared RLX-*b*NPs possess acceptable colloidal stability owing to proper electrical layer (Das et al., 2012).

In the formulation, glyceryl distearate (Precirol ATO 5) serves as a lipid matrix, Carbopol 940 functions as a bioadhesive material, and TGPS plays the role of apportion-promoting enhancer. These three materials were adopted on purpose to construct bioadhesive nanocarrier based on their respective functions. ATO 5 is a solid lipid above the body temperature that can guarantee high drug entrapment and sustained drug release (Xing et al., 2018). Carbomers with a brand name Carbopol, are synthetic macromolecular polymers based on acrylic acid that are crosslinked with either allyl sucrose or allyl ethers of pentaerythritol. Upon contact with liquid, carbomers become hydrated and swelling, and can interact with mucoproteins in the mucus and adhere to the mucosa (Chawla & Saraf, 2012), thus providing bioadhesion. TPGS is a versatile biomaterial that can be used as emulsifier, drug solubilizer, absorption enhancer, and as a modifier for lipid-based drug delivery systems (Yang et al., 2018). In this study, we integrated these functional materials into the formulation of nanoparticles in an attempt to collaboratively enhance the oral delivery efficacy.

### 3.2. *In vitro* drug release

**Figure 3.**
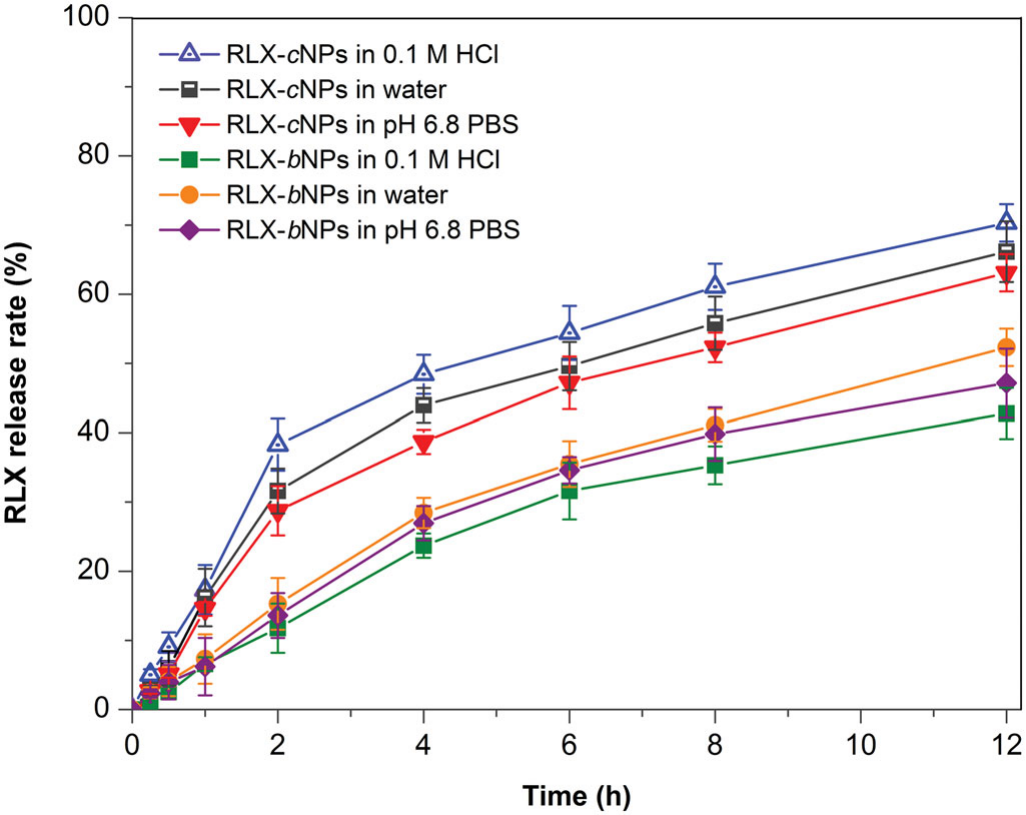
*In vitro* release profiles of RLX from RLX-cNPs and RLX-bNPs in the media of 0.1 M HCl, water, and pH 6.8 PBS. Data expressed as mean ± SD (*n*= 3).

The *in vitro* release profiles of RLX from RLX-*c*NPs and RLX-*b*NPs are shown in Figure 3. RLX-*c*NPs and RLX-*b*NPs exhibited different release profiles. RLX release from RLX-*c*NPs was obviously faster than from RLX-*b*NPs in three media. We assumed that this was attributable to the distinct structure of nanoparticles. RLX-*b*NPs contained a partial proportion of bioadhesive carbomers. This material becomes swelling and glutinous after adsorbing water that can form a diffusion layer, resulting in slowdown of drug release. In addition, the release pattern of RLX-*c*NPs was different from RLX-*b*NPs in terms of different release media. RLX-*c*NPs showed the fastest RLX release in the medium of 0.1 M HCl. This can be explained by the chemical nature of RLX, which is a weak base after desalting that has high solubility in the acidic medium, so the release of RLX from RLX-*c*NPs in 0.1 M HCl was relatively fast compared to other two media. However, the reverse event has taken place in the case of RLX-*b*NPs. RLX-*b*NPs exhibited the slowest drug release in 0.1 M HCl. We think it has something to do with protonization of carbomers in the presence of H^+^ that compacts the diffusion layer. In three media, the accumulative release of RLX-*b*NPs did not exceed 42% within 8 h, a time approximate to the gastrointestinal transport. Anyhow, the drug release of RLX-*b*NPs was relatively slow where no burst release occurred. It has been reported that RLX·HCl is difficultly encapsulated into nanocarriers due to amphiphobicity (Ye et al., 2016; Shah & Rajput, 2018). In this study, we achieved a stable encapsulation of RLX into nanoparticles via *in situ* desalting. *In vitro* release study indicates that RLX would not leak quickly from nanoparticles. The sustained release allows the majority of RLX molecules to be entrapped in the nanoparticles as transporting across the gastrointestinal tract, which is advantageous to RLX absorption via intact nanoparticles. The *in vitro* release profiles of RLX-*b*NPs follow the first-order kinetic process (*R*^2^=0.9562), which demonstrating a diffusion-controlled release along with the devised nanocarrier (Fu & Kao, 2010; Yin et al., 2017b).

### 3.3. Bioavailability enhancement

**Figure 4.**
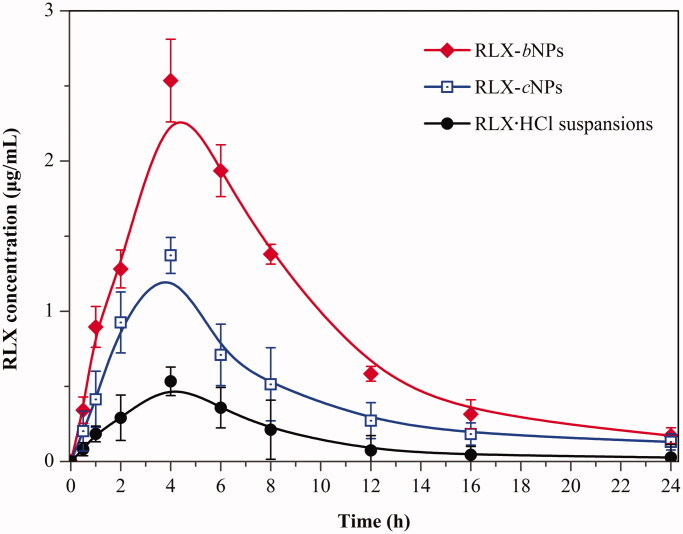
*In vivo* pharmacokinetic curves of plasma RLX concentration versus time after oral administration of RLX·HCl suspensions, RLX-cNPs and RLX-bNPs at a dose of 10 mg/kg (*n*= 6).

The pharmacokinetic profiles of RLX suspensions, RLX-*c*NPs and RLX-*b*NPs after oral administration to rats are shown in Figure 4. The main pharmacokinetic parameters processed by non-compartment model are listed in Table 1. RLX·HCl was poorly absorbed with a suspension formulation. The peak plasma concentration (*C*_max_) was just 0.534 μg/mL. This may be concerned with ionization of RLX·HCl, since ionic drugs are generally difficult to transport across the epithelial membrane. As formulated into lipid nanoparticles, the intestinal absorption of RLX was greatly improved as reflected by RLX-*c*NPs. The *C*_max_ increased to 1.372 μg/mL. For one thing, RLX·HCl being transferred into base in RLX-*c*NPs is favorable for intestinal absorption (Yin et al., 2017a); for another lipid nanoparticles may facilitate the intestinal transport of the cargo by the lymphatic route (Burra et al., 2013). Compared to RLX-*c*NPs, RLX-*b*NPs resulted in higher blood drug levels and absorption extent. The *C*_max_ rose to 2.536 μg/mL, and the area under the plasma concentration versus time curve (AUC_0–_*_t_*) also increased several times. The oral bioavailability of RLX-*b*NPs was calculated to be 556.9% relative to RLX·HCl suspensions, while it was merely 244.7% with respect to RLX-*c*NPs. From the viewpoint of *T*_max_ (the time to *C*_max_), the absorption phase of RLX-*b*NPs was longer than that of RLX-*c*NPs, which could be ascribed to increased intestinal retention of RLX-*b*NPs due to good mucoadhesion. Likewise, the mean residence time (MRT) of RLX-*b*NPs was longer than that of RLX-*c*NPs. These results turn out that RLX-*b*NPs can prolong the absorption duration of RLX and thus enhance oral bioavailability thereof.

**Table 1.** Comparative pharmacokinetic parameters of RLX in rats after oral administration of RLX·HCl suspensions, RLX-*c*NPs, and RLX-*b*NPs (*n*= 6).

Preparation	RLX·HCl suspensions	RLX-*c*NPs	RLX-*b*NPs
*C*_max_ (μg/mL)	0.403 ± 0.182	1.030 ± 0.385	1.995 ± 0.562**
*T*_max_ (h)	3.570 ± 0.653	3.449 ± 0.458	4.049 ± 0.275*
AUC_0–_*_t_* (μg/mL*h)	3.872 ± 1.221	9.591 ± 1.723	21.562 ± 2.321**
MRT (h)	7.141 ± 0.338	6.902 ± 0.686	8.102 ± 0.774*
RBA	–	244.7%	556.9%**

MRT: mean residence time; RBA: relative bioavailability calculated based on one-compartment model; ANOVA, *p< .05, **p< .01, compared with RLX-*c*NPs and RLX·HCl suspensions.

The parent compound of RLX·HCl is a weak base and has poor solubility in water. In order to improve its water-solubility, RLX is mostly made into hydrochloride. However, the hydrochloride form not only tends to ionize, but also reduces its solubility in lipid materials, which causes troubles to develop oral nanomedicines. In the present work, we simultaneously desalinated RLX·HCl using an alkalic cosolvent upon preparing nanoparticles. The resulting bioadhesive nanoparticles (*b*NPs) demonstrated stable entrapment to RLX and exhibited enhancive bioavailability. In addition to providing bioadhesion, Carbopol 940 can also sustain the drug release. It works together with TPGS to increase absorption of RLX. TPGS is frequently used in drug delivery systems as an absorption enhancer (Guo et al., 2013). TPGS has been proven to be promising as P-gp inhibitor, solubilizer/absorption, and permeation enhancer in oral drug delivery. In a recent study, Salah et al. prepared microsponges gels containing TPGS, sodium deoxycholate, and Carbopol 974P for virginal drug delivery of miconazole (Salah et al., 2018). This system utilizes the bioadhesion of carbomers and the absorption-promoting effect of TPGS, improving the mucosal retention of the formulation and the antibacterial activity of the drug. RLX is predominantly used to treat the postmenopausal osteoporosis and estrogen-dependent breast cancer via oral administration. Owing to high presystemic clearance, the oral bioavailability of RLX is reported to be not more than 2%. It is difficult to develop stable oral liquid formulations due to poor solubility both in water and oil, besides vesicles-based gels for transdermal drug delivery (Waheed et al., 2019). Although lipid-based formulations have been investigated for oral delivery of RLX·HCl (Ravi et al., 2014; Shah et al., 2016), these studies ignored the issues of drug/lipid expulsion and storage stability. To overcome drug precipitation from nanoparticles, water-free self-nanoemulsifying drug delivery systems (SNEDDS) (Elsheikh et al., 2012; Jain et al., 2018), electro-sprayed nanoparticles (Varshosaz et al., 2018), and lyophilized solid lipid nanoparticles (Burra et al., 2013) were developed for oral delivery of RLX·HCl. In addition, mesoporous carbon nanospheres were proposed to load RLX·HCl via the adsorption mechanism for bioavailability improvement (Ye et al., 2016). Compared with those systems abovementioned, the bioadhesive system constructed by us exhibits advantages in drug entrapment stability, convenience for use, and absorption enhancement. Enhancement in bioavailability of RLX-*b*NPs can be attributed to the integrated bioadhesion and absorption-promoting effect that prolong the intestinal retention of carriers and facilitate the intestinal absorption.

### 3.4. Cellular trafficking

**Figure 5.**
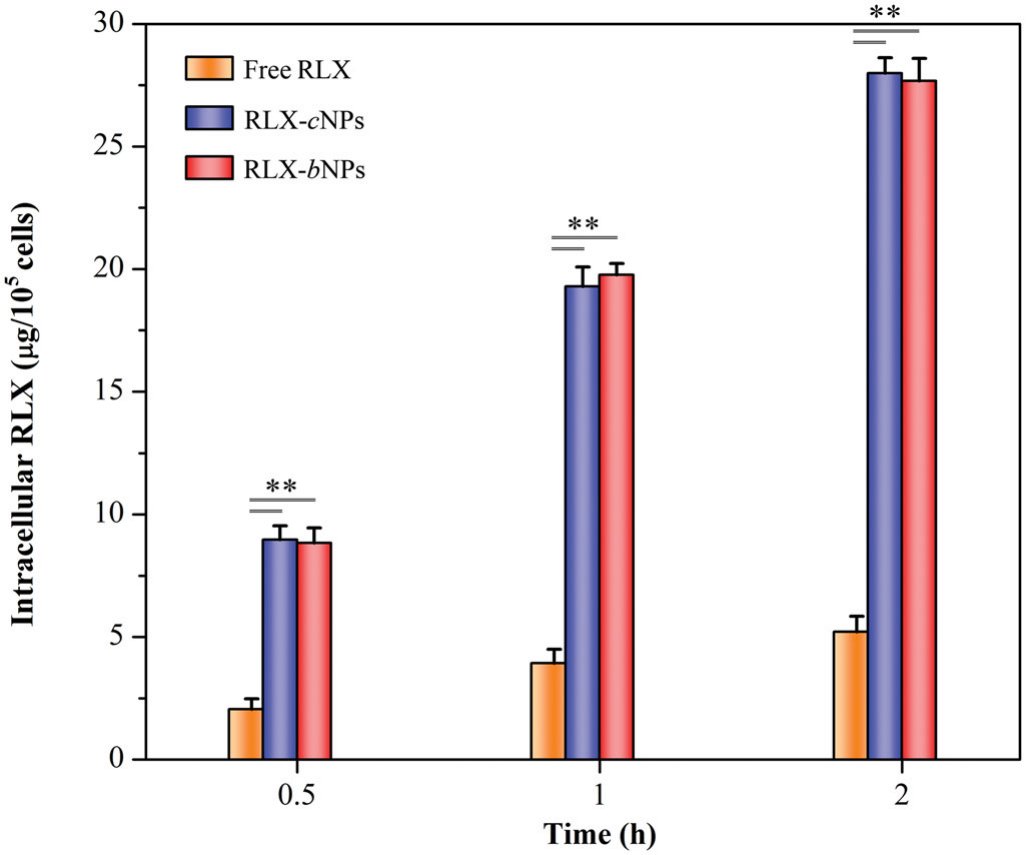
Cellular uptake of free RLX, RLX-cNPs, and RLX-bNPs in Caco-2 cells at the drug concentration of 50 μg/mL. Data shown as mean ± SD (*n*= 3), paired-*t* test, *p*< .01, significantly different compared with free RLX.

The intercellular RLX levels after incubation with free RLX, RLX-*c*NPs, and RLX-*b*NPs were exhibited in Figure 5. Free RLX, in the form of RLX·HCl solution, exhibited the lowest cellular uptake at all investigated time points, which can be ascribed to its ionization in aqueous medium that causes difficulty in transport across the cell membrane. The cellular uptake of RLX significantly increased when it was loaded in nanoparticles as a base. Both RLX-*c*NPs and RLX-*b*NPs resulted in incremental cellular uptake levels compared to free RLX. According to the results of *in vitro* release, it can be known that less RLX would be released from nanoparticles within 2 h. Therefore, the enhancive cellular uptake of RLX is assumed to be related to the overall transport of nanoparticles. Of note, there was no significant difference in cellular uptake between RLX-*c*NPs and RLX-*b*NPs. RLX-*b*NPs use Carbopol 940 in addition than RLX-*c*NPs in the formulation. However, carbomers mainly bind with mucin (glycoproteins) in the mucus (Bera et al., 2016), which have no propensity to adhere to the cell membrane. Hence, it is easily understood that the cellular uptake of RLX-*b*NPs is comparable to RLX-*c*NPs.

**Figure 6.**
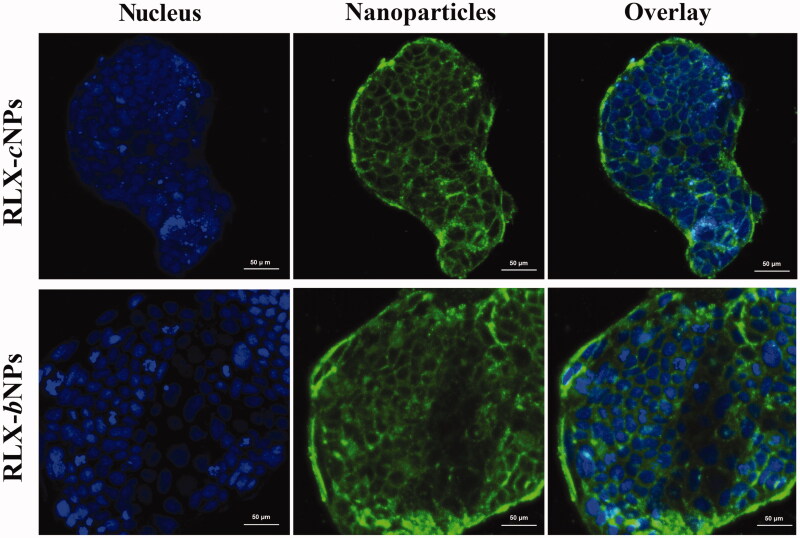
Cellular internalization of RLX-cNPs and RLX-bNPs characterized by CLSM. Nanoparticles labeled by DiO; cell nucleus dyed with Hoechst 33258.

The cellular internalization of RLX-*c*NPs and RLX-*b*NPs was visualized through CLSM and presented in Figure 6. After incubation for 0.5 h, RLX-*c*NPs and RLX-*b*NPs considerably penetrated into the cell colony along the paracellular route, indicating that both lipid-based nanocarriers had high affinity to enterocytes. The cell staining seemed to be more intense in the case of RLX-*b*NPs, but there is no comparability between them. In a short time, nanoparticles have internalized into the cytoplasm, even into the nucleus. Normally, transcellular and paracellular routes are the exclusive pathways of nanoparticle internalization (Murugan et al., 2015). From the CLSM micrographs, it can be seen that the paracellular route dominates the internalization of RLX-*c*NPs and RLX-*b*NPs. Of course, we cannot rule out the involvement of transcellular route. The cellular uptake and internalization results reveal that RLX-*b*NPs are provided with preferable cellular trafficking capacity.

### 3.5. Bioadhesion

**Figure 7.**
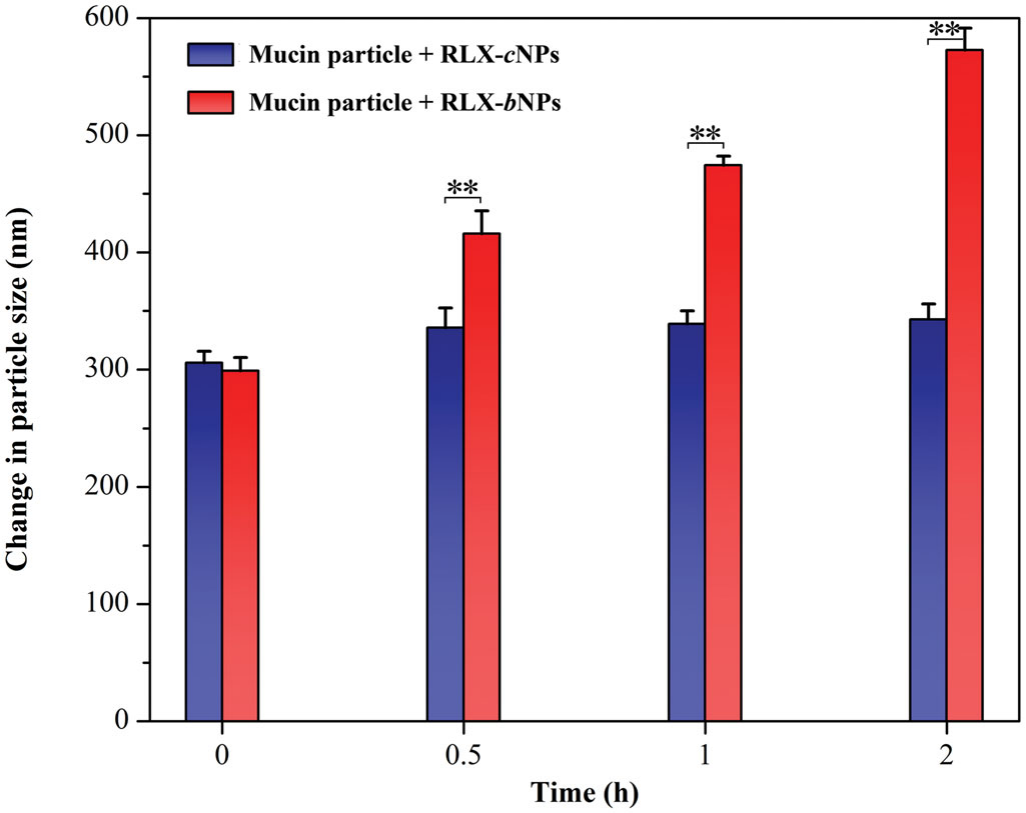
Size evolution of mucin particle upon mixing with RLX-cNPs and RLX-bNPs. Paired-*t* test, *p*< .01, statistically different from each other (*n*= 3).

The changes in the system containing mucin particles upon incubation with RLX-*c*NPs and RLX-*b*NPs are shown in Figure 7. The particle size of mucin system did not change apparently when incubated with RLX-*c*NPs, where the particle size merely increased a few tens of nanometers after incubation for 2 h. In comparison with the initial stage, the *ζ* potential of the system also underwent less change (data not shown). However, in the case of RLX-*b*NPs, the particle size of the system gradually increased as the incubation time extended. The resultant *ζ* potential of mucin system shifted to −48.6 mV from −36.2 mV, which can be attributed to the negative charge of Carbopol polymer. These results strongly supported that mucin particles were highly adhered by RLX-*b*NPs owing to fine bioadhesion, rather than RLX-*c*NPs. When the mucin particles are mingled with nanoparticles with an adhesive property, the charged particles will adsorb onto the mucin particles that results in increase in particle size and changes in *ζ* potential (Wan et al., 2020). It is a practicable *in vitro* testing method to evaluate the mucoadhesive property of a drug delivery system.

**Figure 8.**
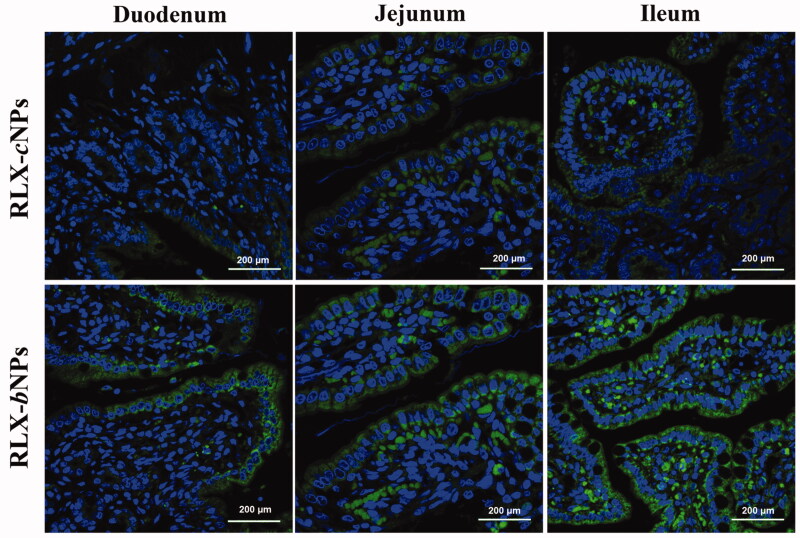
*Ex vivo* imaging on the intestinal retention of RLX-cNPs and RLX-bNPs. Excellent intestinal retention of RLX-bNPs supported by the striking fluorescence remained in the absorptive epithelium after transport for 2 h.

The intestinal permeability of nanoparticles can also be appreciated by their infiltration across the intestinal epithelium. Figure 8 reveals the fluorescence distributions associated with RLX-*c*NPs and RLX-*b*NPs in different absorptive epithelia after administration for 1 h. The basolateral side and the apical side are clearly visible from the longitudinal section after nucleus staining by DAPI. It could be observed that RLX-*c*NPs exhibited weaker infiltration into the intestine as indicated by the fluorescence intensity. The fluorescent staining in the ileum, the main site for absorption of substances, was also not prominent in the case of RLX-*c*NPs. It suggests that the epithelial penetrability or affinity of RLX-*c*NPs is relatively inadequate. As far as RLX-*b*NPs concerned, intense fluorescence staining took place through the whole intestine, especially in the ilium. RLX-*b*NPs were largely concentrated within the microvilli of the intestinal epithelium. Some nanoparticles have successfully penetrated into the central lacteal. RLX-*c*NPs and RLX-*b*NPs exhibit different intestinal mucoadhesion and permeability. As known, good bioadhesion can increase the contact chance and prolong the retention time of the payload on the absorptive epithelia that is beneficial for subsequent penetration (Reineke et al., 2013). This is also the underlying mechanism that RLX-*b*NPs are provided with superb intestinal absorbability and result in enhanced bioavailability.

## 4. Conclusions

In this study, a bioadhesive polymer–lipid hybrid nanosystem was constructed and its suitability as oral delivery carrier of RLX was evaluated. It was shown that carbomer-based *b*NPs possessed excellent mucoadhesive property in comparison with common lipid nanoparticles (*c*NPs). Cellular uptake and internalization analysis proved that RLX-*b*NPs were readily assimilated by Caco-2 cells. Furthermore, the *ex vivo* imaging on transepithelial transport revealed that RLX-*b*NPs went with strong intestinal adhesion and permeability. Enhanced bioavailability of RLX was accomplished through *b*NPs by virtue of good affinity to enterocytes. This work provides fundamental insight into the use of a bioadhesive system for oral delivery of amphiphobic drugs to improve their oral bioavailability.
